# Fermi calculations enable quick downselection of target genes and process optimization in photosynthetic systems

**DOI:** 10.1093/plphys/kiaf103

**Published:** 2025-03-20

**Authors:** Ratul Chowdhury, Wheaton Schroeder, Debolina Sarkar, Niaz Bahar Chowdhury, Supantha Dey, Rajib Saha

**Affiliations:** Department of Chemical and Biological Engineering, Iowa State University, Ames, IA 50011, USA; Voiland School of Chemical Engineering and Bioengineering, Washington State University, Pullman, WA 99164, USA; International Flavors and Fragrance Inc., Palo Alto, CA 94304, USA; Department of Chemical and Biomolecular Engineering, University of Nebraska-Lincoln, NE 68588, USA; Department of Chemical and Biological Engineering, Iowa State University, Ames, IA 50011, USA; Department of Chemical and Biomolecular Engineering, University of Nebraska-Lincoln, NE 68588, USA

## Abstract

Understanding how photosynthetic organisms including plants and microbes respond to their environment is crucial for optimizing agricultural practices and ensuring food and energy security, particularly in the context of climactic change and sustainability. This perspective embeds back-of-the-envelope calculations across a photosynthetic organism design and scale up workflow. Starting from the whole system level, we provide a recipe to pinpoint key genetic targets, examine the logistics of detailed computational modeling, and explore environmentally driven phenotypes and feasibility as an industrial biofuel production chassis. While complex computer models or high-throughput in vivo studies often dominate scientific inquiry, this perspective highlights the power of simple calculations as a valuable tool for initial exploration and evaluating study feasibility. Fermi calculations are defined as quick, approximate estimations made using back-of-the-envelope calculations and straightforward reasoning to achieve order-of-magnitude accuracy, named after the physicist Enrico Fermi. We show how Fermi calculations, based on fundamental principles and readily available data, can offer a first-pass understanding of metabolic shifts in plants and microbes in response to environmental and genetic changes. We also discuss how Fermi checks can be embedded in data-driven advanced computing workflows to enable bio-aware machine learning. Lastly, an understanding of state of the art is necessary to guide study feasibility and identifying key levers to maximize cost to return ratios. Combining biology- and resource-aware Fermi calculations, this proposed approach enables researchers to prioritize resource allocation, identify gaps in predictions and experiments, and develop intuition about how observed responses of plants differ between controlled laboratory environments and industrial conditions.

## Teaser

Fermi calculations derived from domain expertise can yield key insights in photosynthetic systems across lab and industrial settings by gauging metabolic shifts and guiding process optimization.

Advances box

- Recent advances in multitissue modeling, enzyme assays, and enzyme design enable cross-scale calculations and modeling single-protein, single-cell, multicellular, photosynthetic systems (microbes and plants, alike).
- Engineering photosynthetic systems, such as creating crops and microbial strains that are resilient to physical, chemical, and biological stresses and adversaries, have failed to show the sought-after robustness.
- Integration of scales and resources required will start with back-of-the-envelope (Fermi) calculations or estimates. Such rapid, rough, order-of-magnitude quantitative estimates are useful for first-pass analyses prior to committing resources.
- We propose considerations for cross-scale Fermi calculation for resources for experimental and computational studies in terms of compute and cost.

## Introduction

**Figure 1.**
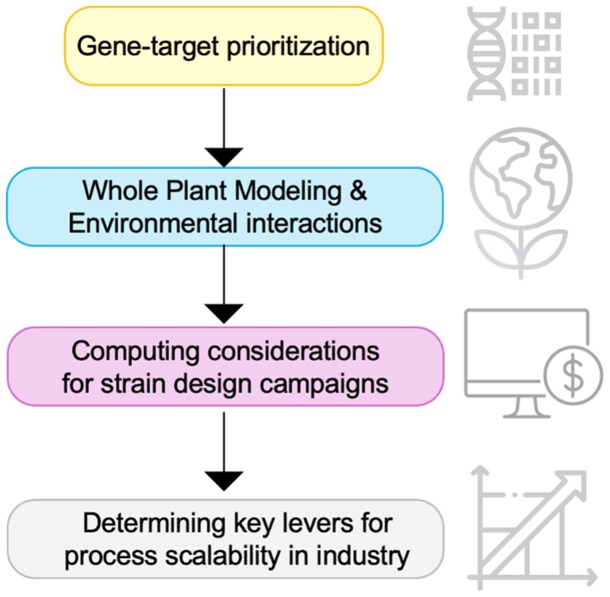
Highlights of this focus article. Herein, we provide several Fermi calculations at key stages for optimizing photosynthetic bio-chasses.

Plant behavior is a complex interplay of biological processes that operate across multiple scales. At the cellular level, enzymes dictate the speed of biochemical reactions, which in turn impact metabolite concentrations and fluxes. These cellular activities contribute to multicellular tissue functions, leading to physiological responses in the whole plant (Fendt et al. 2010; Zhao et al. 2020, 2021). These responses are then further modulated by environmental factors, influencing plant growth, adaptation, and interactions with the surroundings (Mareri et al. 2022). While these biological processes are deterministic in nature, predicting plant behavior remains challenging. The limitations of experimental and computational tools introduce uncertainty, especially when considering interactions across different scales (Tomkins 2023). Similar conclusion can be drawn for photosynthetic microbes (e.g. cyanobacteria; Berla et al. 2013) and anoxic soil microbe including *Rhodopseudomonas palustris* (Brown et al. 2022b) although these represent much simpler unicellular systems. Fermi calculations are rough quantitative estimates using approximate values and simple reasoning to quickly gauge the order of magnitude of a problem, or even the reliability of a state-of-the-art method. By simplifying complex systems into manageable approximations and assumptions, Fermi estimations provide rapid, order-of-magnitude insights and estimates of uncertainty of lab-scale experimental/computational protocols (Oliveira-Filho et al. 2024). This practical approach helps navigate decision-making in both research and agriculture (Fig. 1), thus offering a valuable, science-based tool for optimizing microbial strain engineering, agricultural practices, and enhancing crop performance, even amidst the complexities of the biological systems. We also introduce the concept of Fermi checks, i.e. domain-knowledge-driven simple, yet insightful checks integrated as internal controls at various steps in a workflow. We show how a Fermi check can enable better data-driven, bio-aware machine learning models.

We suggest (i) considerations for across-the-scale lab-based methods and (ii) a Fermi calculation for resources both in terms of compute and cost for a priori gauging of metabolic shifts (i.e. alteration in levels of metabolites) in photosynthetic species owing to nutrient-deficient microenvironment from the single enzyme level (protein structure). Then, it extends to single-cell metabolism (equally applicable for photosynthetic microbes and plant cell-types), followed by multicellular tissue phenotype, and finally to whole plant. Understanding how these systems respond to their environment is crucial for optimizing agricultural practices, ensuring food security, and/or fuel production. These useful first approximations and order-of-magnitude insights leverage a combination of back-of-the-envelope calculations, domain knowledge, and modern computing tools to gain insights into their metabolism under varying environmental conditions.

### The power of simple estimates

**Figure 2.**
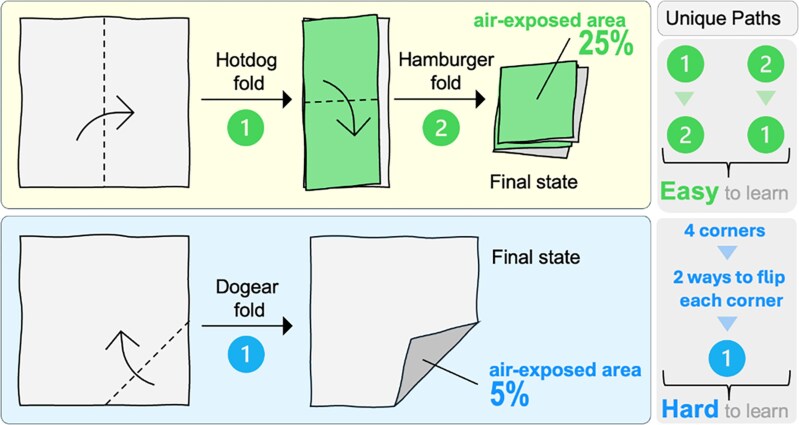
The process of folding a piece of paper in halves twice leads to 1 out of 4 quadrants (25%) of air-exposed surface. This can be achieved through 2 operations and along 2 paths of making a hotdog and a hamburger fold. As this has 4 sets of information (2 operations; 2 paths), it is quite easily learnable. However, a simple but arbitrary dog-ear fold (1 operation; 8 paths) is almost impossible to replicate with fidelity. This is a case where a data-driven technique will engage significantly higher compute resources to learn the description of the apparently simple dog-ear fold. However, Fermi estimates of air-exposed surface areas of the bottom face equal to 25% and 5%, respectively, intuitively convey that the first case is a squarely folded paper and the second one only has a tiny fold at the edge.

Fermi calculations offer an invaluable tool in biological research by allowing for quick estimates of metabolic responses and gauging uncertainties of standard practices by leveraging domain expertise. These preliminary estimations guide further investigations by highlighting potentially larger effects, optimizing resource allocation in research projects. This targeted approach ensures that efforts are concentrated on the most promising avenues, potentially leading to quicker breakthroughs (Oliveira-Filho et al. 2024). Furthermore, when these back-of-the-envelope calculations deviate from observed results, it signals potential knowledge gaps in our understanding. This knowledge gap analysis acts as a springboard for future research, prompting further experimentation and theoretical exploration, enabling researchers to navigate complex data sets with greater clarity and insight. As an analogy with origami, we show 2 paper folding experiments (Fig. 2) to highlight that the amount of information being processed is not the true indicator of the complexity of the system. In such cases, a simple yet precise metric obtained through Fermi calculations can lead to better and faster understanding of the underlying process.

### Prioritization of gene targets when engineering photosynthetic systems

**Figure 3.**
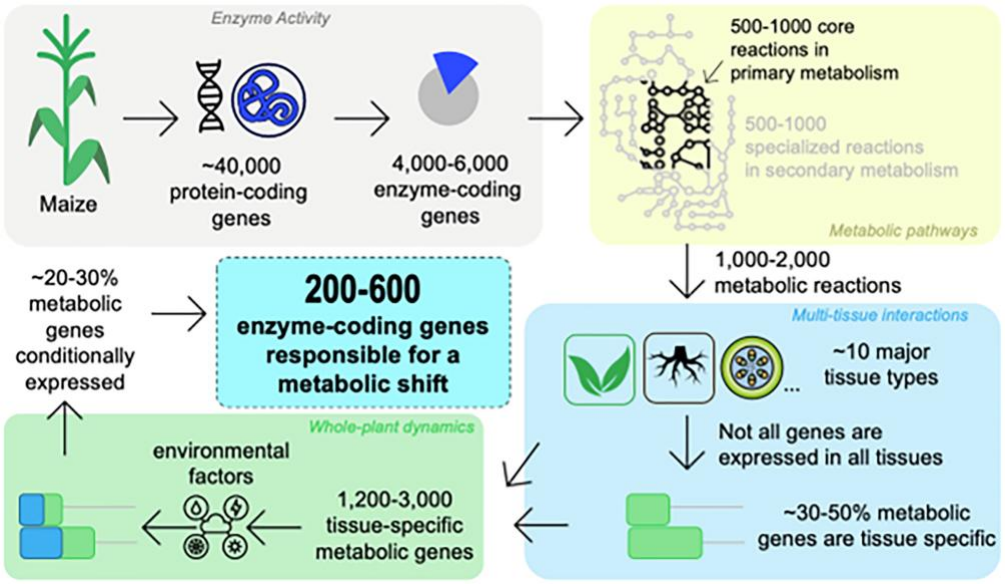
Overview of a Fermi calculation to pinpoint how many enzyme-coding genes are likely to be responsible an environmentally driven metabolic shift in maize. Such calculations span scales of genome, enzyme, pathway, tissue, to whole plant. Numbers have been estimated from several references cited above.

A multiscale approach (see Fig. 3) to understand environment-driven crop (such as maize) phenotypes typically starts at the genomic level (Chen et al. 2023), which translates to the amount and activity of different biocatalytic enzymes within each cell (Ma et al. 2017). Enzyme activity gatekeeps differential utilization of metabolites through a portfolio of careful biochemical conversions (Seaver et al. 2015; Noor et al. 2025) that catabolize nutrients and anabolize smaller pieces to generate necessary molecules for life. These molecules (metabolites) shuttle not only across intracellular compartments, but also across cell types and tissues, which act as translocating chemical signals (Griffiths et al. 2016). By looking across these different biological hierarchies, it is possible to discern the mechanistic basis of a given phenotype.

A multiomics workflow integrates annotated genomes, transcriptomics, and proteomics to map enzymatic genes to tissue-specific reactions, filtering by phenotypic changes and prioritizing target genes for further testing under control vs. perturbed conditions (Fig. 3). In a plant such as maize, such an approach can wean down the list of target genes from >40,000 to <600. This is the final set of high-confidence genes to be further validated using in vitro experiments, or feed into a high-throughput experimental or computation workflow to explore combinatorial effects (Chowdhury et al. 2014; Love et al. 2014).

Additionally, we included a brief description of how each level informs the whole plant behavior—(i) *Protein structure and enzyme activity*: Knowledge of protein structure databases (experimental Protein Data Bank (Zardecki et al. 2022), deep learning structure predictors AlphaFold2 (Jumper et al. 2021) using sequence alignments, ESMFold (Lin et al. 2023), and RGN2 (Chowdhury et al. 2022b) from single sequences) enables estimates of enzyme activity under varying nutrient conditions (Wittig et al. 2012; 2018). Changes in macroenvironment (such as heat or nitrogen stress) translate to changes in microenvironment of the salts that are about these enzymes (Zhang et al. 2020). These consequently lead to altered activities. (ii) *Single-cell metabolism*: Building on enzyme activities, we can estimate changes in metabolic flux through key pathways within a single plant cell as some biochemical conversions are now expedited or impeded. This provides insights into how individual cells adapt or manifest external changes. (iii) *Multicellular tissue phenotype*: The collective response of cells within a tissue allows tracking tissue development (root growth, leaf size, etc.), based on the underlying metabolic shifts. Thus, shuttling of multiple metabolites across tissues leads to concerted changes in the plant phenotype. Similar considerations apply while identifying gene targets for metabolic engineering of photosynthetic microbes (Berla et al. 2015; Lin et al. 2017; Alsiyabi et al. 2021; Brown et al. 2022a).

### Considerations for artificial intelligence/machine learning usage to design robust transgenic photosynthetic organisms through in silico enzyme engineering

Artificial intelligence/machine learning (AI/ML) can play a significant role in discovering novel enzymatic routes, kinetic parameter estimation, and metabolic modeling for air–plant–soil ternary interaction. Paradigms of AI/ML include (ii) predictive modeling, (ii) virtual screening, (iii) molecule optimization, (iv) simulation of biological pathways, and (v) integration with high-throughput screening and reinforcement of design-build-test-learn cycles (Wang et al. 2024). Predictive AI models can predict the nutritional value and/or toxicity of novel pesticide or growth-augmenting molecules, where ML models trained on existing data could be used to identify promising de novo enzymatic pathways, albeit often without any true biological causation (Mazurenko et al. 2020). Virtual in silico screening can thereafter leverage AI to rapidly rank large pathway libraries to pinpoint those with desired biochemical traits such as high drought tolerance and cost-effectiveness in terms of on-farm application and scale up. Molecule optimization can be used to fine-tune/tailor promising enzyme candidates from preliminary rounds of in vitro screening for target phenotypes (such as food quality and biofuel production). To this end, generative models, when trained with sufficient bio-awareness of enzymatic biology, will hold the key to de novo pathway discovery and empowering existing design tools (e.g. NovoStoic (Kumar et al. 2018)). This will enable prediction of soil microbe symbiotic relationships with plants. We provide realistic cost estimates of each step for efficient design of photosynthetic strains.

### Considerations for enzyme activity assessment in vitro, in vivo, and in silico

**Figure 4.**
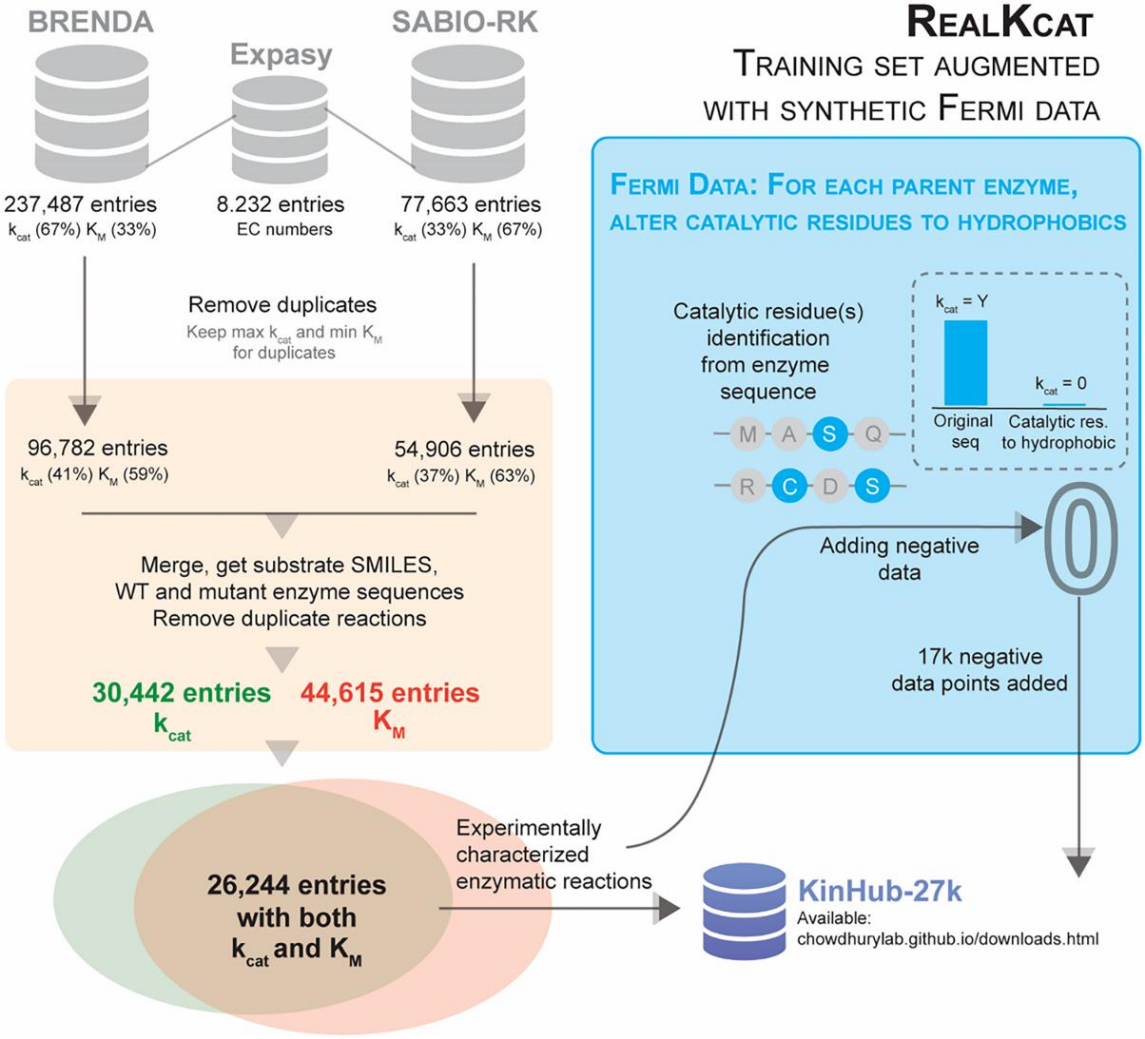
Intuitive Fermi checks can bolster high-performance computing. Above is the workflow where we demonstrated a simple Fermi check on experimental enzyme kinetics data sets to add ∼17,000 synthetic negative data points—thereby adding bio-awareness to our data-driven, machine learning framework (RealKcat). This enhances model accuracy toward predicting an enzyme's catalytic activity/efficiency.

Functional enzymes are currently the state-of-the-art biomolecular workhouse for biomanufacturing regardless of their usage (Boukid et al. 2023). However, design of novel enzymes and tailoring existing ones require robust characterization workflows. Depending on whether in vitro and in vivo characterization of an enzyme is intended, they come with unique considerations. In vitro assessments permit a controlled environment where specific variables can be manipulated, leading to a clear understanding of enzyme kinetics, substrate specificity, optimum activity parameters, and many others (Bonowski et al. 2010). In vitro studies miss the complexity of cellular and systemic contexts, leading to oversights when predicting in vivo activity. Posttranslational modifications, enzyme compartmentalization, and the influence of cellular environments (i.e. macromolecular crowding) play significant roles in actual enzyme function but are often not replicated in vitro studies (García-Contreras et al. 2012). In vivo enzyme assessments, thus, display the natural physiological environment but with little control over experimental design. Further, many systems display high natural variability in enzyme expression and function (e.g. the role of single nucleotide polymorphisms SNPs (Sarkar and Maranas 2020). Due to high variability and complexity, a more physiological reflective method, in vivo enzyme assessments, and tissue-level regulation occlude mechanistic insights into enzyme activity (Ou et al. 2018). Although integrating current advanced in vivo techniques (biomarker analysis, spatial transcriptomics, and metabolomics) helps bridge the gap between in vitro results and physiological reality, they require comprehensive knowledge bases of the portfolio of enzyme variant activity in a living organism (Nam et al. 2024). While this has prompted a push for ML-based predictors for enzyme kinetic parameters from sequence and substrate information, these tools are still catalytically unaware and at best map sequence similarity to kinetic parameter similarity. Point mutations to key catalytic residues on the enzyme show marginal to no changes to predicted catalytic activities while we expect complete abolition of any catalytic ability (Ribeiro et al. 2020). A simple Fermi check suggests creating enzyme sequences with catalytic residues replaced by aliphatic groups should result in destroying catalytic turnover. In our recent work, we added this synthetic data set to demonstrate significantly improved model performance and catalytic awareness (Sajeevan et al. 2025) (Fig. 4).

## Considerations in multitissue, whole plant genome-scale models

Information about catalytic activities of key enzymes affiliated to specific metabolic pathways is necessary prior to understanding cellular behavior through genome-scale models (GSMs). However, preliminary efforts assume all enzymes to be at full catalytic efficiency, thereby casting it as purely network problem which enforces mass and charge balance across molecular “making and breaking” reactions. The first multiorgan plant GSM was reconstructed for barley (Grafahrend-Belau et al. 2013), subsequently for barrelclover (Pfau et al. 2018), *Arabidopsis* (Shaw and Cheung 2018; Schroeder and Saha 2020), soybean (Moreira et al. 2019), foxtail millet (Shaw and Cheung 2019), and rice (Shaw and Cheung 2021). These models, while useful to characterize interorgans crosstalk under various conditions, did not have all 4 major organs of a plant—root, stalk, leaf, and seed. The reconstructed iZMA6517 (Chowdhury et al. 2023) to this end is the largest multiorgan maize genome-scale metabolic model (GSM) for the B73 genotype, based on earlier root (Chowdhury et al. 2022a) and leaf (Simons et al. 2014) GSMs and newly reconstructed stalk and kernel GSMs, linked through vascular tissues. For all the GSMs, thermodynamically infeasible cycles (TICs) were resolved using the OptFill (Schroeder and Saha 2020) pipeline. Overall, iZMA6517 includes 6,517 genes, 5,228 unique reactions, and 3,007 unique metabolites, with 2,305 reactions, 2,405 metabolites, and 6,285 genes shared across all organs. Next, upon contextualization with heat and cold stress transcriptomics data, iZMA6517 accurately predicted the plant growth rate patterns, including the individual organs, accurately under both stress conditions. Moreover, applying metabolic bottleneck analysis on iZMA6517 revealed thermodynamic driving force, energy production, and reducing power generation axis as the major determinant of the plant phenotype under heat and cold stress conditions. Thereby, iZMA6517 can be used to accurately predict plant phenotype under diverse stress conditions (Chowdhury et al. 2023).

However, reconstructing comprehensive multitissue models presents significant challenges, particularly in terms of the cost, planning, and resource allocation for both in silico and in planta experiments. Developing such models requires extensive omics data collection across different tissues, involving high-throughput omics technologies across various phases of plant growth—an effort that is both expensive and time-consuming. Moreover, integrating different omics data sets to accurately represent tissue-specific metabolic activities requires careful selection of appropriate omics data integration algorithms. Experimental validation adds another layer of complexity, as it involves in planta studies that are logistically demanding and resource-intensive and often require a multiyear timeframe. These challenges underscore the importance of strategic resource allocation and interdisciplinary collaboration in effectively reconstructing and refining multitissue GSMs. Unlike microbial community modeling, which focuses on the interactions between multiple microbial species within a shared environment, multiorgan plant modeling must account for the diverse and interconnected metabolic activities across different tissues within a single organism.

### Pan-species, generalizable Fermi calculations for whole-plant carbon assimilation and drought phenotypes

Here, we will use drought-stressed tree species as an example of Fermi calculations as applied to environment-driven phenotypes: trees are of interest as mechanisms of long-term carbon sequestration, as well as potential bioenergy crops (Barbosa et al. 2024), though the basic principles outlined here should be applicable to most plant species. In modeling plants under drought condition, simultaneous integration of multiple physiological, metabolic, and transcriptomic changes (many from basic biology) is key and often overlooked. That is, generally 1 or 2 such factors are incorporated, rather than a combination of all 3, as was first done (to our knowledge) by a recent (2024) GSM of poplar (Barbosa et al. 2024). In plants, drought results in multiple systemic effects. The first category of effects is those related to water conservation. Perhaps the most obvious method of water conservation is the more frequent complete or partial closure of stomata (Cominelli et al. 2010). This comes with the side effect of reduced carbon dioxide assimilation. Recalling the basic photosynthetic carbon fixation equation (below), as carbon dioxide assimilation decreases, through decreased stomatal conductance compounded with lower water availability, so does the yield of the plant.


(1) $$6\;CO_{2}+6\;H_{2}O\to C_{6}H_{12}O_{6}+6O_{2}$$

The carbon fraction in plant biomass can range from 18.4% to 75% by weight (USDA Forest Service 2022), with a rule of thumb being that woody plant biomass is about 50% carbon (USDA Forest Service 2023), which is a recommended conversion by the Intergovernmental Panel on Climate Change (IPCC) (Penman et al. 2003) and is similar to estimates for softwood ($0.51\;\;g\;C\;g^{-1}\;\mathrm{DW}$) and hardwood ($0.49\;g\;C\;g^{-1}\;\mathrm{DW}$) species (Telmo et al. 2010; Petráš et al. 2021). Assuming carbon content in plant biomass does not vary dramatically under drought conditions, plant mass yield should be linearly related to carbon assimilation, which is in turn linearly related to stomatal conductance (as conductance is an upper bound on $CO_{2}$ uptake). Carbon assimilation, stomatal conductance, and carbon sequestration have been evaluated in several tree species. In Norway spruce, it was demonstrated that carbon sequestration, as biomass, decreases 67% in a drought year, compared to control (Martínez-Sancho et al. 2022). In an analysis of 20 broadleaf evergreen tree and shrub species, the ratio of drought-stressed stomatal conductance to unstressed ranged between 2% and 40% (Marchin et al. 2021). For poplar and olive trees mild, moderate, and severe drought stress, carbon assimilation can decrease by approximately 50%, 75%, and 90%, respectively (Bogeat-Triboulot et al. 2007). In deciduous hardwood trees, chronic drought stress led to a 41% decrease in annual carbon sequestration (Brzostek et al. 2014). Therefore, in drought-stressed tree species, biomass carbon, and therefore biomass, decreases significantly. This decrease is exacerbated by the severity of drought, and we suggest that 50%, 75%, and 90% as useful Fermi approximations of biomass yield decrease under mild, moderate, and severe drought stress.

Further, this decrease is unevenly distributed across tissues, with stem, leaf, flowers (Choi et al. 2023), and reproductive biomass fractions decreasing and root mass fractions increasing (Eziz et al. 2017). In one published meta-analysis (Eziz et al. 2017), root mass fraction generally increased approximately 10% in drought-stressed plants, compared to a 2% to 5% decrease in leaf and 4% to 8% decrease in stem, with the most variability in the change in reproductive tissue biomass ranging from approximately a 3% to 12% decrease. Another meta-analysis of 128 studies identified that under drought conditions root to shoot mass ratio increased by an average of about 14%, mostly due to increased root diameter at the cost of length and length density (Zhou et al. 2018). In poplar for instance, this appears due to the maintenance of root growth through mild and moderate drought and the steep decline in plant height and diameter growth rates (Bogeat-Triboulot et al. 2007). Combined with drought stress-induced limitations on total plant biomass yield, above ground mass (AGM, consisting of leaf, stem, and reproductive tissues) yield estimates can be further decreased an additional 10% (at least) to approximately 45%, 68%, and 81% of unstressed yields.

For estimating mass of carbon sequestration, rather than yield, one common measure of trees can be used, namely, diameter at breast height (DBH). From forestry research, a simple linear regression can be used to reliably estimate root system biomass from DBH, as shown below which is a relationship derived for European evergreen and deciduous trees (Drexhage and Colin 2001).


(2) $$\log\;(M_{\mathrm{root}})=2.44\log\;(\mathrm{DBH})-1.56$$

Where $M_{\mathrm{root}}$ is the mass of the root system in kilograms and DBH is the diameter of breast height in meters ($R^{2}=0.94,\;P<1E-4$). Under drought conditions, we propose that root mass estimates may be increased by 10% to account for the higher biomass allocation to roots in these conditions. Similarly, AGM also correlates well with DBH. Using the Comprehensive Database of Diameter-based Biomass Regressions for North American Tree Species, total above ground biomass may be estimated as follows for hardwood and softwood trees (Jenkins et al. 2004).


(3) ln(AGM)=2.4ln(DBH)−2.2

where AGM is in kilograms and DBH is in centimeters. The relationship parameters are the median values from both hard and softwood trees, rounded to the nearest tenth for convenience. This was done as the values were largely similar, with the slope having a standard deviation 0.07 and the intercept having a standard deviation of 0.24. These correlations are quite strong, with $R^{2}$ values between 0.938 and 0.988 (Jenkins et al. 2004), which is more than sufficient accuracy for first-pass calculations. Due to shift in tissue mass allocation in drought conditions, we propose that AGM may be scaled to 90% in Fermi calculations. Further, this same resource gives simple relations for estimating tissue ratios (Jenkins et al. 2004), though these coefficients of determination are much lower than for AGM ($R^{2}\leq 0.256$ for the relations given). Two important ratios for hardwood and softwood foliage as foliage limits photosynthetic rate (putting a total cap on carbon metabolism) can be written in Fermi-level simplicity as follows (Jenkins et al. 2004):


(4) $$\mathrm{Hardwood}:\;\ln\;\left(\frac{M_{\mathrm{leaf}}}{\mathrm{AGM}}\right)=-4.1+\frac{5.9}{\mathrm{DBH}}$$

(5) $$\mathrm{Softwood}:\;\ln\;\left(\frac{M_{\mathrm{leaf}}}{\mathrm{AGM}}\right)=-3.0+\frac{4.5}{\mathrm{DBH}}$$

where $M_{\mathrm{leaf}}$ is the estimated leaf tissue mass and parameters are rounded to the nearest tenth for simpler Fermi limits. In a model built on 26 plant species, the maximum potential photosynthetic rate is estimated as between 0.1 and $9.8\;\mathrm{mmol}\;C\;g^{-1}\;\mathrm{leaf}\;h^{-1}$, with a median of $0.7\;\mathrm{mmol}\;C\;g^{-1}\;\mathrm{leaf}\;h^{-1}$ ($700\;\mathrm{mmol}\;C\;kg^{-1}\;h^{-1}$) (Kikuzawa and Lechowicz 2006). Therefore, considering equation (3) on AGM and equation (4) or (5) as appropriate, Fermi calculations of carbon assimilation rate can be made and scaled by approximate leaf mass to estimate limits on carbon availability to plant metabolism. If we wish to create a single equation to translate DBH to maximum photosynthetic potential (combining equations (3) and (4) or (3) and (5)),


(6) $$\mathrm{Hardwood}:P_{\max}=700\exp\;\left(2.4\;\ln\;(\mathrm{DBH})-6.3+\frac{5.9}{\mathrm{DBH}}\right)$$

(7) $$\mathrm{Softwood}:P_{\max}=700\exp\;\left(2.4\;\ln\;(\mathrm{DBH})-5.5+\frac{4.5}{\mathrm{DBH}}\right)$$

where $P_{\max}$ is the maximum photosynthetic potential (in mmol C h^−1^). While equations (6) and (7) seem somewhat more complex, these are powerful calculations, identifying maximum metabolic capacity from a single field measurement. Further, we can expand this to a per-day estimate recognizing that there is roughly 12 to 14 h of daylight, so the calculation coefficient can be scaled appropriately. Finally, we can take advantage of climatic data sets to estimate the growing season. In the United States, this can be done by retrieving data from the Agricultural Applied Climate Information System (AgACIS, agacis.rcc-acis.org/), which can be used to retrieve growing seasons by state and county where data is available. For example, consider poplar trees, a hardwood species native to the Pacific Northwest with Washington State University managed plantations in Othello, Washington (Adams County) (Costantini et al. 2018). From the AgACIS, the growing season (above 32 °F) is 168 days. Therefore, using equation (6), assuming a young tree at this location with a DBH of 6 cm, with an average of 13 h of daylight, the maximum photosynthetic fixation of carbon over the course of 1 year would be approximately $553\;\mathrm{mol}\;C\;\mathrm{yea}r^{-1}$. It has also been noted that between 30% and 60% of carbon assimilated is lost by respiration (Amthor et al. 2019). Assuming a 50% loss, a reasonable maximum total carbon sequestration by this hypothetical tree would be $276\;\mathrm{mol}\;C\;\mathrm{yea}r^{-1}$, or a total of $3.3\;\mathrm{kg}\;C\;\mathrm{yea}r^{-1}$. Given that tree biomass can be assumed as approximately 50% carbon, and assuming carbon as the limiting nutrient, this hypothetical tree would grow by about $6.6\;\mathrm{kg}\;\mathrm{yea}r^{-1}$. The 6.6 kg carbon per year estimate provides a benchmark for photosynthetic efficiency in trees, which is crucial for understanding carbon sequestration potential. This value helps inform GSMs and contributes to designing and scaling up efficient photosynthetic organisms. These GSMs will then be able to estimate photosynthetic limitation under drought-mediated stresses. These estimates all come from publicly available data and a single measurement, DBH; therefore, we suggest that in cases where a limited number of measurements can be made, DBH is a useful and key Fermi metric for estimating tree mass, tissue yields (under drought and normal conditions), maximum photosynthetic capability, and yearly maximum carbon. In combination with a drought monitoring category (viz. drought.gov) converted into a mild (D_0_), moderate (D_1_), or severe category (D_2_ through D_4_), the appropriate (aforementioned) carbon assimilation scaling may be selected and many key insights into the state and capabilities of a tree can be quickly estimated. While such a key metric such as DBH might not exist for herbaceous species, less precise estimates could be used. Exemplars of these alternate metrics focus on whole plant and tissue mass outcomes, photosynthetic efficiency, drought effects, and net carbon loss to respiration.

### Fermi calculation for estimating photorespiration and wax, hormone, and RUBISCO production levels in drought-resistant crops

Another water-saving effort is the increased production of wax, particularly cuticular wax, to reduce transpiration and aid in the production against high temperatures (Xue et al. 2017). Cuticular wax may be comprised of alkanes, alcohols, fatty acids, terpenes, esters, aldehydes, triterpenoids, and sterols (Wu et al. 2023), many of which are metabolically expensive carbon-containing compounds representing another drought-induced diversion in carbon metabolism. Since wax loading is not well-studied in tree species, here we use mulberry trees as an example, where total wax yield is 248 *µ*g dm^−2^ (2.48E-2 $g\;m^{-2}$) (Ni et al. 2015). Leaf mass area (LMA, that is grams of leaf biomass per $m^{2}$) can vary by an order of magnitude in forests (Poorter et al. 2009). Using temperate forests as our basis of calculation with LMA 81 $g\;m^{-2}$ (Fermi estimate), leaf wax load accounts for <0.03% of leaf biomass. Increased wax production is likely decoupled from plant yield and hence ignored in Fermi calculations. Similarly, while drought-associated plant hormones such as GABA, abscisic acid, jasmonic acid, and gibberellins would be expected to have increased accumulation under drought conditions (Yoshida and Fernie 2024), hormone concentrations are generally low. For example, in drought-stressed poplar trees, abscisic acid content rises from a base level of about $400\;\mathrm{ng}\;g^{-1}\;\mathrm{FW}$ to $1,600\;\mathrm{ng}\;g^{-1}\;\mathrm{FW}$ (Popko et al. 2010). This indicates a 4-fold increase in abscisic acid content, and therefore, a Fermi check will constitute ascertaining a similar 4-fold increase through the upstream methyl erythrose phosphate (MEP) pathway in vascular plants (Milborrow 2001). An ancillary category of effects are metabolic diversion of energy and carbon flow, aside from wax and hormone production. First, photorespiration rates increase. In ambient growth conditions, it is estimated that approximately 25% of photosynthetic energy is lost due to RUBISCO oxygenase activity (Abogadallah 2011). For example, photorespiration increased by approximately 31% in olive trees (Boussadia et al. 2008) and by 200% to 300% in drought-stressed *Lotus corniculatus* (herbaceous plant). In addition, RUBISCO has a notably low $k_{\mathrm{cat}}$ value, in the range of 0.6 to 13 $\mathrm{mol}\;CO_{2}\;\mathrm{mo}l^{-1}\;\mathrm{sites}\;s^{-1}$ (2,160 to $46,800\;\mathrm{mmol}\;CO_{2}\;\mathrm{mmo}l^{-1}\;\mathrm{sites}\;h^{-1}$) (Sage 2002). To sustain photosynthetic rates presented in the previous section for the hypothetical poplar tree, at 253 $\mathrm{mmol}\;CO_{2}\;h^{-1}$, would require approximately 0.0054 to 0.12 mmol of RUBISCO active sites. Assuming one active site per enzyme complex, and a molecular weight of 81.2 kDA (NCBI reference sequences XP_002313525 and XP_024445660), this hypothetical tree would require between 0.44 and 9.5 g of RUBISCO at any time (assuming saturation kinetics of $v\approx k_{\mathrm{cat}}[E]$). From equation (4), this hypothetical tree would have about 362 g of leaves, and therefore, RUBSICO would account for between 0.12% and 2.6% of total leaf biomass (probably on the higher end due to low $CO_{2}$ saturation), which is well in line with other estimates (2% in trees) (Bar-On and Milo 2019). RUBISCO's inefficiency therefore has led to numerous attempts to improve photosynthetic efficiency through RUBISCO engineering, elsewhere noted as “probably the holy grail of green biotechnology” (Peterhansel and Offermann 2012) given the obvious scale of the problem highlighted by Fermi calculations such as these.

## Cost considerations GPUs in AI training and inference based on modeling framework choice

Graphical processing units (GPUs) accelerate AI computations through parallel processing, with high-end models like NVIDIA A100 featuring 512 tensor cores for efficient matrix operations. However, GPU performance is often constrained by memory bandwidth and data transfer speeds. Training large language models like GPT-3 can cost between $500,000 and $4.6 million and months of runtime on GPU clusters (Li 2020). It is important to weigh cloud services against in-house data servers based on cost despite the leaps of AI/ML in biology (viz. Nobel Prize in Chemistry) as accurate protein function prediction for industrial, pharmaceutical, and engineering tasks remains unsolved. GPU selection depends on factors like training, inference needs, memory requirements, and latency constraints (see Tables 1 and 2). While hosted model services (e.g. Hugging Face) offer a low-cost, scalable option for many startups, those requiring fine-grained control, or secure access may need to build their own GPU infrastructure. Cloud providers offer flexibility and lower upfront costs, but large-scale, regulated operations may benefit from on-premises data servers. These numbers (Table 1) can be connected to the focused set of enzymes (Fig. 3) to evaluate overall project costs.

**Table 1.** Estimated compute and experimental costs for understanding chemically induced metabolic shifts. The compute costs have been estimated based on facility user fees, from simulations run on NVIDIA A100 GPUs installed on NOVA High-Performance Compute at Iowa State University and cloud instances done on Amazon Web Services

Category	Cost component	Estimated cost per molecule basis
**Compute costs**		
Model training	GPU hours (on-site at Iowa State)	$80
Virtual screening	Cloud computing costs (Amazon)	$10
Data storage and management	Storage and database maintenance	$2
**Experimental costs**		
Synthesis of molecules	Chemical synthesis	$100
High-throughput screening	Biological assays and testing	$200
Validation studies	In vivo testing on plants	$300
Total estimated cost		$692

**Table 2.** Rough estimates of compute costs for a single project (3 to 5 years duration) studying ∼200 enzyme–molecule pair interactions

Step	Description	Estimated GPU power (TFLOPS)	Estimated cost (USD)
Train autoencoder	Encode 770,000 molecules (MOSES) using autoencoder	High (10 to 100+)	High ($50,000 to $100,000)
Reinforcement learning for reward function or diffusion models for molecule generation	Optimize reward function for atomic arrangements, or predict novel atomic arrangements	Medium (1 to 10)	Medium ($2,000 to $4,000)
Protein–molecule docking	Simulate docking poses for each molecule–protein pair	Medium (1 to 10)	Medium ($3,000 to $5,000)
All-atom MD simulations (500 ns)	Simulate molecule–protein dynamics for 500 ns each	Very high (100+)	Very high ($10,000+)
Replica exchange MD (100 ns)	Simulate dynamics at varying temperatures (100 ns)	High (10 to 100+)	High ($10,000 to $15,000)
Clustering and ranking	Group and rank molecules based on binding score	Low (<1)	Low (<$3,000)
Picking top interactions	Select candidates for experimental validation	N/A	N/A

The compute costs have been estimated using projections from on-site NVIDIA A100 GPU, 64 GB memory node, 8 processor code/node at Iowa State, and the MOSES molecule database.

High-end GPUs like NVIDIA's A100 and H100 are ideal for training large models, while less powerful GPUs can suffice for smaller models or less latency-sensitive applications.

Pre- and postprocessing steps usually require minimal GPU power. The estimated GPU power and cost requirement are broad ranges and depend on factors like (i) molecule complexity, (ii) sparsity of data sets (i.e. how many of similar molecule clusters exist in the database), (iii) protein size, (iv) number of known homologs or size of training set, and (v) dynamic simulation parameters (timesteps, coarse graining) and desired simulation accuracy. The estimated costs are ballparked on cloud computing instances with commercial GPUs and typical simulation times for biomolecular systems. Actual costs may vary depending on provider, instance type, and software used.

### Simple Fermi calculation for calculating kilowatt-hour of power consumed to train and host enzyme activity predictor models

Most generative AI models are trained to be hosted open source for the community to use. A Fermi estimate for resource consumption, for a GPT-3-scale model for enzyme activity prediction and de novo enzyme sequence generation, includes training and inference runs.

*Step 1:* Estimating the number of GPUs used for GPT-3-type protein language model trained on protein sequences (250 m sequences from UniProt and their structures from ESM Atlas)

While the exact number of GPUs used to train GPT-3 is not publicly disclosed, we can make a reasonable estimate based on available information and industry practices. For our calculation, we will use the following:


$$N_{\mathrm{GPU}}=1,024V100\mathrm{GPUs}.$$

This estimate aligns with the scale of resources typically required for training large language models like GPT-3.

*Step 2:* Calculating kilowatt-hour units burnt for GPT-3 training

Assuming a training time of 48 h (2 days; like original GPT-3 model) and power consumption of 0.4 kW per V100 GPU,


$$\mathrm{kWh}\_\mathrm{burn}t_{\mathrm{training}}=N_{\mathrm{GPU}}\times kW_{\mathrm{per}\_\mathrm{GPU}}\times \mathrm{training}\;\mathrm{hours}$$

$$\mathrm{kWh}\_\mathrm{burn}t_{\mathrm{training}}=1,024\times 0.4\times 48\approx 19,661\mathrm{kWh}$$

*Step 3:* Calculating expected kilowatt-hour units burnt for inference in 1 year

Given that the PDB database is accessed ∼5 m accesses per day (https://www.rcsb.org/pages/about-us/index), we assume this model will be accessed at least 0.5 m times per day with each inference taking 0.1 s.


$$\begin{array}{r} H_{i}=(500,000\times 365\times 0.1)/3,600\approx 5,000\;\mathrm{hours}\;\mathrm{of}\;\mathrm{inference}\;\mathrm{per}\;\mathrm{year} \end{array}$$

$$\mathrm{kWh}\_\mathrm{burn}t_{\mathrm{inference}}=N_{\mathrm{GPU}}\times 0.0001\times kW_{\mathrm{per}\_\mathrm{GPU}}\times H_{i}$$

Here, 0.0001 is a scaling factor that represents the reduced computational load during inference compared to training, as inference does not require gradient calculations and parameter updates. We assume that per-inference consumes 1/10,000th of the computational power needed for training (Snell et al. 2024).


$$∴\mathrm{kWh}\_\mathrm{burn}t_{\mathrm{inference}}=1\times 0.0001\times 0.4\times 5,000\approx 2\mathrm{kWh}\;\mathrm{per}\;\mathrm{year}\;(\mathrm{negligible}\;\mathrm{compared}\;\mathrm{to}\;\mathrm{training})$$

So, the total kilowatt-hour units burnt per training cycle and across inference cycles would be approximately 20,000 kWh based on these example values. US Energy Information Administration (EIA) confirms that this is close (∼200%) to the average annual US household power usage (10,500 kWh).

This Fermi calculation provides a way to estimate the energy consumption based on the number of GPUs, model hyperparameters, and usage hours. Overall, we draw out key considerations in term of knowing gaps in state-of-the-art ML models and making Fermi estimates on resources for understanding the metabolic shifts in photosynthetic systems across the scales of single enzymes, to cells, colonies, and across multiple tissues. We also provide Fermi resource estimates for in silico, in vitro, and in vivo experiments.

## Current practices of Fermi calculations in industry on photosynthetic microbes

Fermi calculations are often used in the industry across the entire manufacturing pipeline. In the very initial conceptualization stages of a project, market-driven forecast analysis is used to determine margins, vet competitors, and predict market share. This is used to back-calculate capital expenditure, research, and development timelines and assess feasibility of current technology vs. project success needs. These, in turn, strongly dictate the required downstream production yield, research and development resources needed, and recovery and formulation efficiency.

### Quality of experimental measurements

High-throughput screening assays are a staple workhorse in industry. This approach is widely applicable and scalable, enables rapid hypothesis generation and testing, and provides immediate targets for successive design-build-test-learn cycles. To increase resolution and usability, it is beneficial to maximize data collected for each variant tested. These data sets can be rationally designed to increase the overall phenotypic information gleaned and enable quick quality assurance and quality control (QA/QC). To that end, Fermi calculations are useful for constructing internal controls in the data collection campaign. For example, during a photosynthetic strain engineering campaign, growth rate can be used to estimate the expected number of proteins per cell (Milo 2013). This can then be used as a benchmark to evaluate different proteomics data collection methods and sample prep protocols. Going further, different variants can also be compared in a similar fashion to determine the impact of strain engineering interventions on cellular protein synthesis and whole plant phenotypes.

### Fermi calculation to determine scalability of engineering of photosynthetic microbes for biofuel

Biotechnology has conventionally relied on using bacterial and fungal hosts as cell factories. This is due to ease of genetic transformation (Cho et al. 2022), growth on relatively cheap and abundantly available nutrient sources (Yan et al. 2024), and regulatory laws (FDA 2023). These precision fermentation platforms are steadily replacing conventional chemical manufacturing processes due to being environment friendly and sustainable while meeting cost bottom-lines. In recent years, using photosynthetic microalgae as production hosts is gaining traction, especially in relation to biofuel production. Microalgae can be grown using just sunlight as energy, CO_2_ as carbon, and wastewater effluent as nitrogen and phosphorus sources. In comparison to terrestrial plants, these can grow 10 to 15 times faster, an important consideration for commercialization. However, close considerations must be made when choosing a photobioreactor (PBR) vs. bioreactor as the manufacturing process. Mass transfer, pH control, energy input, productivity gap between lab vs. manufacturing scale, ease of strain engineering, and process improvements to increase yield are some of the pivotal parameters that govern such a choice. Here, we use biofuel production as a case study and evaluate the suitability of algal bioreactors for commercial production.

Their maximal biomass productivity is given as follows:


(8) $$\max\;\mathrm{biomass}\;\mathrm{productivity}=\frac{\left(\mathrm{ASR})\times (\mathrm{PAR})\times (\mathrm{PE}\right)}{\mathrm{Biomass}\;\mathrm{energy}\;\mathrm{content}}$$

where ASR refers to annual solar irradiance (GJ ha^−1^ year^−1^), PAR is photosynthetically active radiation, and PE is photosynthetic efficiency. The theoretical maximum value of PE is 25.9% of photosynthetically active radiation (PAR) (Zhu et al. 2008), but for microalgae is 10.3% PAR (Norsker et al. 2011). The biomass energy content ranges from 20 to 30 (GJ ton^−1^) (Morweiser et al. 2010; Schlagermann et al. 2012). Considering the annual solar radiation to be between 19,800 and 91,700 GJ ha^−1^ year^−1^ and PAR to be 48.7% of total ASR, the maximal algal biomass productivity can be calculated as follows:


(9) $$\max\;\mathrm{biomass}\;\mathrm{productivity}=\;\frac{\left((91,700+19,800)/2)\times (0.487)\times (0.103\right)}{20}=\;140\;\mathrm{ton}\;ha^{-1}\;\mathrm{yea}r^{-1}$$

To compare this to other commercialized biofuel such as ethanol and biodiesel from corn and sugarcane, we need to express this to maximal biofuel productivity as follows:


(10) $$\begin{array}{c} \max\;\mathrm{biofuel}\;\mathrm{productivity}=(\max\;\mathrm{biomass}\;\mathrm{productivity})\\ \times \;(\mathrm{lipid}\;\mathrm{content})\times (\mathrm{energy}\;\mathrm{content})\\ =\;140\;\times \left(\frac{0.25+0.5}{2}\right)\times 0.86=45.15\;\mathrm{TOE}\;ha^{-1}\;\mathrm{yea}r^{-1} \end{array}$$

where the lipid content varies from 25% to 50% (Morweiser et al. 2010; Schlagermann et al. 2012) and energy content is 0.86 (ton of oil equivalent [TOE] ton^−1^). To determine the economics behind the process, we can use the maximal algal biofuel productivity and plug in expenses (divided into capital and operating) to determine the cost of producing 1 L of biofuel as follows:


(10) $$\mathrm{total}\;\mathrm{cost}=\frac{\left(\frac{\mathrm{capital}}{\mathrm{ROI}}\right)+(\mathrm{operating})}{\max\;\mathrm{biofuel}\;\mathrm{productivity}}$$

where ROI is the return on investment on the capital invested. Huntley and Redalje (2007) reported a 940,000 USD ha^−1^ capital expense for PBRs and operating expense of 15 USD ha^−1^ year^−1^. Considering a 5-year ROI, the total cost of algal biofuel would be as follows:


(11) $$\begin{array}{r} \mathrm{total}\;\mathrm{cost}=\frac{\left(\frac{940,000}{5}\right)+(15)}{45.15}=\;4,164\;\mathrm{USD}\;\mathrm{TO}E^{-1}=4.164\;\mathrm{USD}\;L^{-1} \end{array}\;\;\;$$

Compared to this, commercial biofuels being sold today which are made from conventional energy crops cost much lesser at 0.21 to 0.99 USD L^−1^. This simple Fermi calculation indicates that algal bioreactors are not a good substitute to current biotechnological methods of biofuel production. Although cost prohibitive by today's technology standards, this framework can help locate key areas for future investment. Improving the lipid content of biomass, creating cheaper materials for PBR and improving their efficiency, and modifying the underlying photosynthetic apparatus (Berla et al. 2013, 2015; Lin et al. 2017) are some areas where innovation can help drive downstream feasibility.

Consequently, the true power of Fermi estimates lies in combining collective domain knowledge to intuitive biological realities to enable meaningful data-driven synthetic biology.

Outstanding questions box

- Can artificial intelligence and machine learning models be informed by Fermi calculations to have impact in multiscale integration of in vitro, in vivo, and in silico efforts?
- How can the use of Fermi calculations aid in the designs of such efforts, including identifying necessary resources (compute and cost), and to filter out what is unnecessary?
- How can researchers select the genes, enzymes, proteins, metabolites, pathways, and environmental factors most key to integration of multiscale dynamics in photosynthetic systems and reliable recapitulation of observed phenotypes?
- How can researchers use Fermi calculations and expert intuition to carefully select priors to tune multiscale photosynthetic system behavior with appropriate resource expenditures (both compute and direct dollars)?

## Data Availability

All data discussed in this study have been directly referenced. No additional data is linked to the manuscript.

## Dive Curated Terms

The following phenotypic, genotypic, and functional terms are of significance to the work described in this paper:

carbon CHEBI: CHEBI:27594

FDA Gramene: Fluorescein Diacetate
FDA Araport: Fluorescein Diacetate
